# Statistical methods and software for the analysis of highthroughput reverse genetic assays using flow cytometry readouts

**DOI:** 10.1186/gb-2006-7-8-r77

**Published:** 2006-08-17

**Authors:** Florian Hahne, Dorit Arlt, Mamatha Sauermann, Meher Majety, Annemarie Poustka, Stefan Wiemann, Wolfgang Huber

**Affiliations:** 1 Division of Molecular Genome Analysis, German Cancer Research Center, INF 580, 69120 Heidelberg, Germany; 2 EMBL - European Bioinformatics Institute, Wellcome Trust Genome Campus, Cambridge CB10 1SD, UK

## Abstract

Highthroughput cell-based assays with flow cytometric readout provide a powerful technique for identifying components of biologic pathways and their interactors. Interpretation of these large datasets requires effective computational methods. We present a new approach that includes data pre-processing, visualization, quality assessment, and statistical inference. The software is freely available in the Bioconductor package prada. The method permits analysis of large screens to detect the effects of molecular interventions in cellular systems.

## Abstract 2

A software tool for the analysis of high-throughput cell-based assays is presented.

## Background

Cell-based assays permit functional profiling by probing the roles of molecular actors in biologic processes or phenotypes. They perturb the activity or abundance of gene products of interest and measure the resulting effect in a population of cells [1,2]. This can be done in principle for any gene or combination of genes and any biologic process. There is a variety of technologies that rely on the availability of genomic resources such as full-length cDNA libraries [3-7], small interfering RNA libraries [8-12], or collections of protein-specific interfering ligands (small chemical compounds) [13]. Loss-of-function assays that investigate the effect of silencing or (partial) removal of a gene product or its activity [10] are distinguished from gain-of-function assays, in which the function of a gene product is analyzed after its abundance or activity is increased [14].

Depending on the process of interest, phenotypes can be assessed at various levels of complexity. In the simplest case a phenotype is a yes/no alternative, such as survival versus nonsurvival. More detail can be seen from a quantitative variable such as the activity of a reporter gene measured on a fluorescent plate reader, and even more complex features can involve time series or microscopic images. Although flow cytometry is among the standard methods in immunology, it has not been widely used in high-throughput screening, probably because of the lack of automation in data acquisition as well as in data analysis. However, the technology has evolved significantly in the recent past, and the latest generation of instruments can be equipped with high-throughput screening loaders that permit the measurement of large numbers of samples in reasonable periods of time [15]. One major advantage of flow cytometry is its ability to measure multiple parameters for each individual cell of a cell population. Whereas conventional cell-based assays are limited to recording population averages, this approach allows the investigation of biologic variation at the single cell level.

A broad range of tools is available for analyzing flow cytometry data at a small or intermediate scale [16-18], but there is a lack of systematic computational approaches to analyze and rationally interpret the amount of data produced in high-throughput screens. Here we describe methods and software to fulfill these requirements.

## Results and discussion

We demonstrate our methodology on a dataset that was collected in gain-of-function cellular screens probing for mediators of cell growth and division, in particular using assays for DNA replication, apoptosis, and mitogen-activated protein kinase (MAPK) signaling. The experiments were performed in 96-well microtiter plates in which each well contained cells transfected with a different overexpression construct. Along with the phenotype of interest, the amount of overexpression of the respective proteins was recorded via a fluorescent YFP (yellow fluorescent protein) tag. In the following discussion we refer to one microtiter plate as one experiment.

The flow cytometry data consist of four values for each cell: two morphologic parameters and two fluorescence intensities. The morphologic parameters are forward light scatter (FSC) and sideward light scatter (SSC), and they measure cell size and cell granularity (the amount of light-impermeable structures within the cell). One of the fluorescence channels monitors emission from the YFP tag of the overexpressed protein, whereas the other channel detects the fluorescence of a fluorochrome-coupled antibody. Because many phenotypes are amenable to detection via specific antibodies, this can be considered a general assay design theme that, in principle, is applicable to a wide range of cellular processes.

### Data pre-processing and quality

The pre-processing includes import of the result files from the fluorescence-activated cell sorting (FACS) instrument, assembly and cleaning up of the data, removal of systematic biases and drifts (a process often referred to as 'normalization'), and transformation to a format and scale that is suitable for the following analysis steps. Here we do not deal with the technical aspects of data import and management, and refer the interested reader to the documentation of the software package prada for a thorough discussion of these [19].

#### Selection of well measured cells on the basis of morphology

**Figure 1:**
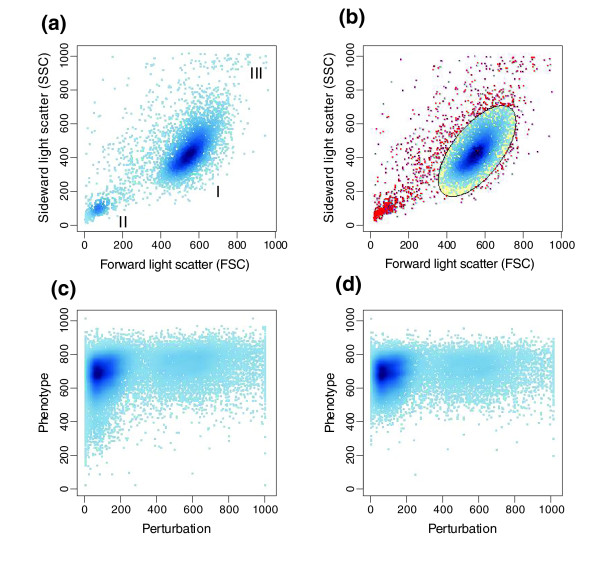
Selection of well measured cells. **(a) **Scatterplot of FACS data showing typical properties of morphologic parameters. FSC corresponds to cell size and SSC to cell granularity. Several subpopulations can be distinguished: (I) healthy and well measured cells, (II) cell debris, and (III) cell conjugates and air bubbles. **(b) **Robust fit of a bivariate normal distribution to the data. The ellipse represents a contour of equal probability density in the distribution and is used as a user-defined cut-off boundary (two standard deviations in this example). Points outside the ellipse (marked in red) are considered contaminants and are discarded from further analysis. Scatterplots of perturbation versus phenotype **(c) **before and **(d) **after removing contaminants. The proportion of outlier data points is reduced significantly. Here, they correspond to measurements with very small phenotype values (cell debris). FACS, fluorescence-activated cell sorting; FCS, forward light scatter; SSC, sideward light scatter.

Most experimental cell populations are contaminated by a small amount of debris, cell conjugates, buffer precipitates, and air bubbles. The design of FACS instruments usually does not allow perfect discrimination of these contaminants from single, living cells during data acquisition, and hence they can end up in the raw data. To a certain extent we can discriminate contaminants from living cells using the morphologic properties provided by the FSC and SSC parameters. The joint distribution of FSC and SSC for transformed mammalian cells typically exhibits an elliptical shape, and most contaminants separate clearly from this main population (Figure 1a). The core distribution of healthy cells is approximated by a bivariate normal distribution in the (FSC, SSC) space, allowing the identification of outliers by their low probability density in that distribution. Thus, measured events that lie outside a certain density threshold can be regarded as contamination. We fit the bivariate normal distribution to the data by robust estimation of its center and its 2 × 2 covariance matrix (Figure 1b). This is appropriate if the cell population is homogeneous, the proportion of contaminants is small, and the phenotype of interest is not itself associated with large changes in the FSC or SSC signal. A rough pre-selection using some fixed FSC and SSC threshold values, as provided by most FACS instruments, further increases robustness.

To see how this affects the data, Figure 1 panels c and d show scatterplots of the two fluorescence channels measuring the perturbation and the phenotype before and after removal of contaminants. We observe a reduction in the proportion of data points with very small fluorescence values in both channels after removing contaminants. This is reasonable because the fluorescence staining is intracellular, and hence cell debris is not expected to emit strong fluorescence. In addition, we have removed some of the data points with very high fluorescence levels, which apparently correspond to cell conjugates.

For our example data it is possible to determine global, experiment-wide parameters of the core distribution of healthy and well measured cells. However, some experimental settings may also demand adaptive estimates, for example if the cell morphology is expected to change as a result of the perturbation (as is the case for apoptotic cells) or if systematic shifts occur during the course of one experiment.

#### Correlation of fluorescence and cell size

**Figure 2:**
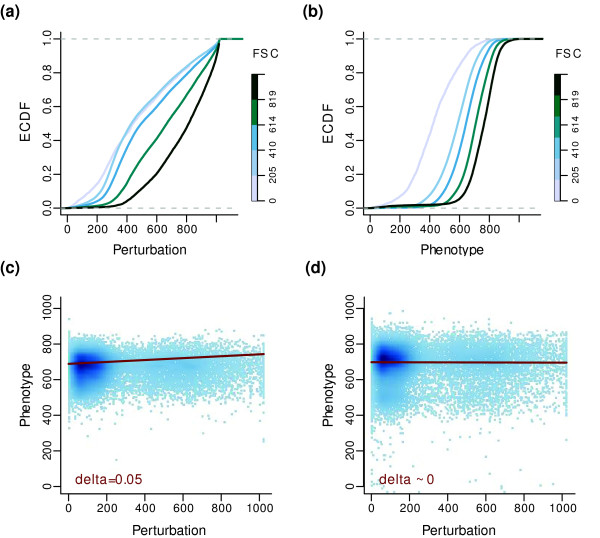
Correlation of fluorescence and cell size. Empiric cumulative distribution functions (ECDF) of fluorescence values for **(a) **perturbation and **(b) **phenotype showing their positive correlation with cell size. The fluorescence values were stratified into subsets corresponding to five quantiles (0-20%, 20-40%, 40-60%, 60-80%, and 80-100%) of cell size (forward light scatter), and the ECDF for each stratum was plotted in a different color. With increasing cell size, an increase in fluorescence values is also observed. **(c) **Regression line fitted to the data showing spurious correlation between the two parameters. In this case, the perturbation is known to cause no phenotype, and hence the correlation is considered to be artifactual. **(d) **After adjusting for cell size, the two parameters are uncorrelated.

Regardless of the presence of fluorochromes, every cell emits light when it is excited by a laser - a phenomenon referred to as autofluorescence. Autofluorescence intensities frequently correlate with cell size, and through this effect often spurious correlations between different fluorescence channels can occur. In our data, the unspecific autofluorescence adds both to the specific fluorescence emitted by the fluorochrome-conjugated antibody measuring the phenotype and to that of the YFP-expressing construct, and it is positively correlated with cell size (Figure 2a,b). This results in an apparent, unspecific increase in the response variable for higher levels of perturbation (Figure 2c). To recover the specific signal we use FSC as a proxy for size, and fit the linear model:

*x*_*total *_= *α *+ *βs *+ *β*_*specific*_   (1)

Where *x*_*total *_is the measured fluorescence intensity, *s *is the cell size as measured by the forward light scatter, *α *and *β *are the coefficients of the model, and *x*_*specific *_is the specific fluorescence. We compute *α *and *β *by robust fit of a linear regression of *x*_*total *_on *s*, and obtain estimates for *x*_*specific *_from the residuals (Figure 2d). This is done for each fluorescence channel individually. The artifactual correlation due to autofluorescence is absorbed by *β*. The parameter *α *absorbs baseline fluorescence, as discussed below.

#### Systematic variation in signal intensities between wells

**Figure 3:**
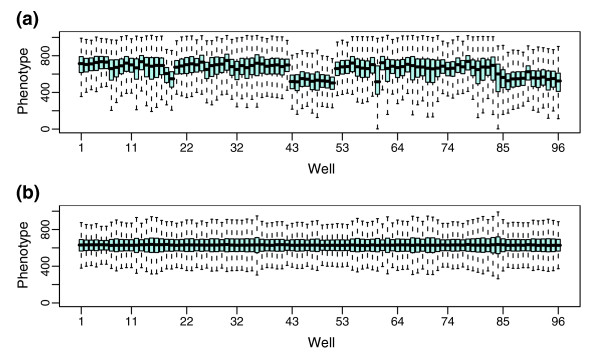
Systematic variation in signal intensities. **(a) **Box plot of raw fluorescence values measuring the phenotype for a 96-well microtiter plate. Differences in the mean values are identified for individual wells, and several wells are affected by a block effect. **(b) **Data after normalization.

In our data we often observe variation in the overall signal intensities for different wells on a microtiter plate (Figure 3a), which may be due to various drifts in the equipment, such as changes in laser power or pipetting efficiencies. Although such effects should ideally be avoided, and large variations should prompt reassessment of the experimental setup, small variations are adjusted by the model described by equation 1. In particular, they are fitted by the intercept term *α*. The biologically relevant information is retained in the residuals. A common baseline of the adjusted values is obtained by adding the mean of *α *averaged over all wells (Figure 3b).

### Statistical inference

Flow cytometry provides individual measurements for each cell of a population, and so we should like to use statistical procedures to model the behavior of the whole population and to draw significant conclusions. Choosing the appropriate statistical model is a crucial step in data analysis because we want it to represent as many features of the data as possible without imposing too many assumptions. For different biologic processes different types of responses can be expected, and so we also need different models. In our data we observe two types of response - binary and gradual.

**Figure 4:**
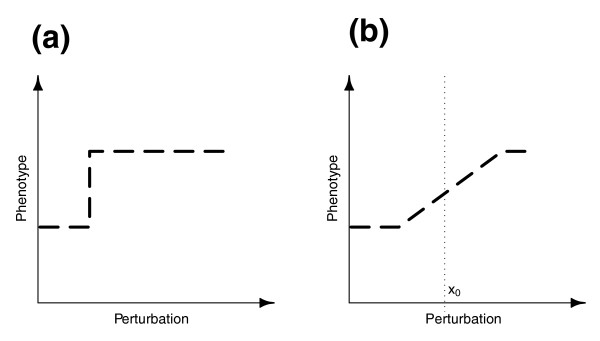
Response types. **(a) **Binary response. Above a certain threshold of perturbation, a discrete phenotype can be observed. **(b) **Continuous response. The effect size of the phenotype correlates with the amount of perturbation. It is typically measured for mild perturbation levels (x_0_).

Many biologic processes can be considered on/off switches in which, after internal or external stimulation above a certain threshold, a distinct cellular event is triggered (Figure 4a). This kind of binary response is typical for apoptosis. One key player of the apoptotic pathway is the enzyme caspase-3, which is activated at the onset of apoptosis in most cell types. Activation is rapid and irreversible, and once the cell receives a signal to undergo apoptosis most or all of its caspase-3 molecules are proteolytically cleaved. This is the point of no return, and all subsequent steps inevitably lead to the death of the cell [20]. Thus, caspase-3 activation is essentially a binary measure of the apoptotic state of a cell. Similarly, cell proliferation is regulated in a binary manner, with cells only progressing further in the cell cycle after reception of appropriate signals.

In contrast, many cellular signaling pathways are continuously regulated. The MAPK pathway, which plays a role in cell cycle regulation, is a prominent example. It consists of several kinases, enzymes with the ability to phosphorylate other molecules, in a hierarchical arrangement. By selective phosphorylation and de-phosphorylation reactions a signal can be passed along the hierarchy [21]. The activity of this pathway can be continuously regulated both in a positive and in a negative manner. So, in contrast to apoptosis and cell proliferation, in which the response is essentially a yes/no decision, here the response is of a gradual nature (Figure 4b).

#### Modeling binary responses

**Figure 5:**
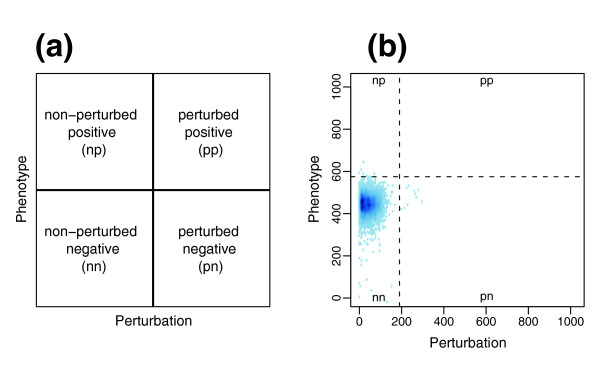
Setup of boundaries. **(a) **Discretization of data showing binary response in four subtypes. **(b) **Mock control used for setup of boundaries.

A natural approach to modeling binary responses is to dissect the data into four subtypes: perturbed versus nonperturbed cells, and cells exhibiting the effect of interest versus nonresponding cells (Figure 5a). Thresholds for this separation can be obtained either adaptively, for each well, or more globally, for the whole plate. Because of the potential problems with over-fitting in the adaptive approach, we choose the latter, making use of the premise that the values of the pre-processed data are comparable across the plate. Figure 5b shows thresholds determined from a high percentile (99%) of the data from a negative control.

An estimator for the odds ratio, a measure of the effect size, is defined by the following equation:

OR=pp+1pn+1⋅nn+1np+1     (2)
 MathType@MTEF@5@5@+=feaafiart1ev1aaatCvAUfeBSjuyZL2yd9gzLbvyNv2Caerbhv2BYDwAHbqedmvETj2BSbqee0evGueE0jxyaibaiKI8=vI8tuQ8FMI8Gi=hEeeu0xXdbba9frFj0=OqFfea0dXdd9vqai=hGuQ8kuc9pgc9s8qqaq=dirpe0xb9q8qiLsFr0=vr0=vr0dc8meaabaqaciGacaGaaeqabaqadeqadaaakeaacaWGpbGaamOuaiabg2da9maalaaabaGaamiCaiaadchacqGHRaWkcaaIXaaabaGaamiCaiaad6gacqGHRaWkcaaIXaaaaiabgwSixpaalaaabaGaamOBaiaad6gacqGHRaWkcaaIXaaabaGaamOBaiaadchacqGHRaWkcaaIXaaaaiaaxMaacaWLjaWaaeWaceaacaaIYaaacaGLOaGaayzkaaaaaa@49E7@

The symbols on the right hand side of equation 2 are defined in Figure 5a. Pseudo-counts of 1 are added in order to avoid infinite values in the case of empty quadrants [22]. It is often convenient to consider the logarithm of the odds ratio, because it is symmetric for upward and downward effects. To test for the significance against the null hypothesis of no effect, we use the Fisher test [23].

**Figure 6:**
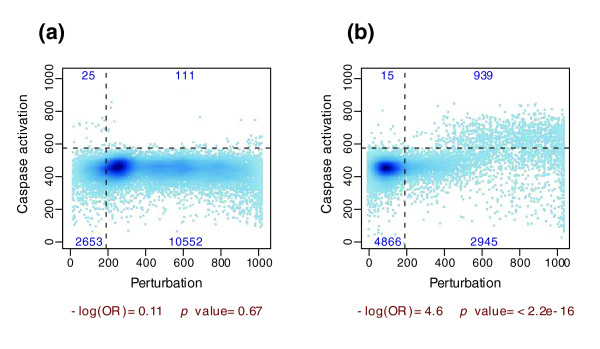
Example results for binary response-type assays from a screen targeting apoptosis regulation. Cell counts for the respective quadrants are indicated on the edges of the plots. **(a) **Non-affector (YFP), with effect size close to zero and insignificant *P *value. **(b) **Activator (Fas receptor), with both large effect size and significant *P *value. OR, odds ratio.

Sample results from a screen aiming to identify activators of the apoptosis pathway are shown in Figure 6. Overexpression of the Fas receptor protein in Figure 6b leads to strong activation of apoptosis, as indicated by both high effect size and a significant *P *value. This is consistent with the cellular role played by the Fas receptor, which mediates apoptosis activation as a consequence of extracellular signaling. Overexpression of the YFP protein in Figure 6a apparently does not affect apoptosis, proving that the activation in Figure 6b is not caused by the fluorescence tag alone.

#### Modeling continuous responses

The gradual nature of these types of responses supports the use of regression analysis. Because the effect may deviate from linearity in the range of perturbations that we observe, we use a robust local regression fit:

*y *= *m*(*x*) + *ε*   (3)

Where *x *is the perturbation signal, *y *is the response, *m *is a smooth function (for example, a piece-wise polynomial), and *ε *is a noise term. We obtain an estimate of *m *from the function locfit.robust in the R package locfit [24]. This also calculates

*δ *= m^(x0)
 MathType@MTEF@5@5@+=feaafiart1ev1aaatCvAUfeBSjuyZL2yd9gzLbvyNv2Caerbhv2BYDwAHbqedmvETj2BSbqee0evGueE0jxyaibaiKI8=vI8tuQ8FMI8Gi=hEeeu0xXdbba9frFj0=OqFfea0dXdd9vqai=hGuQ8kuc9pgc9s8qqaq=dirpe0xb9q8qiLsFr0=vr0=vr0dc8meaabaqaciGacaGaaeqabaqadeqadaaakeaaceWGTbGbaKaadaqadiqaaiaadIhadaWgaaWcbaGaaGimaaqabaaakiaawIcacaGLPaaaaaa@37A7@   (4)

which is a robust estimate of the slope of *m *at the point *x*_0_. *x*_0 _is an assay-wide, user-defined parameter that corresponds to a mild perturbation that does not deviate strongly from the physiologic value. This approach is resistant to nonlinear, biologically artifactual effects caused by perturbations that are too strong, without the need for a sharp cut-off. To obtain a dimensionless measure of *effect size*, we divide

z=δδ0     (5)
 MathType@MTEF@5@5@+=feaafiart1ev1aaatCvAUfeBSjuyZL2yd9gzLbvyNv2Caerbhv2BYDwAHbqedmvETj2BSbqee0evGueE0jxyaibaiKI8=vI8tuQ8FMI8Gi=hEeeu0xXdbba9frFj0=OqFfea0dXdd9vqai=hGuQ8kuc9pgc9s8qqaq=dirpe0xb9q8qiLsFr0=vr0=vr0dc8meaabaqaciGacaGaaeqabaqadeqadaaakeaacaWG6bGaeyypa0ZaaSaaaeaaiiGacqWF0oazaeaacqWF0oazdaWgaaWcbaGaaGimaaqabaaaaOGaaCzcaiaaxMaadaqadiqaaiaaiwdaaiaawIcacaGLPaaaaaa@3D0C@

Where *δ*_0 _is a scale parameter of the overall, assay-wide distribution of *δ*. We use the median absolute value of all *δ *in the assay. A simple measure of the significance against the null hypothesis of no effect is obtained through dividing the estimate m^(x0)
 MathType@MTEF@5@5@+=feaafiart1ev1aaatCvAUfeBSjuyZL2yd9gzLbvyNv2Caerbhv2BYDwAHbqedmvETj2BSbqee0evGueE0jxyaibaiKI8=vI8tuQ8FMI8Gi=hEeeu0xXdbba9frFj0=OqFfea0dXdd9vqai=hGuQ8kuc9pgc9s8qqaq=dirpe0xb9q8qiLsFr0=vr0=vr0dc8meaabaqaciGacaGaaeqabaqadeqadaaakeaaceWGTbGbaKaadaqadiqaaiaadIhadaWgaaWcbaGaaGimaaqabaaakiaawIcacaGLPaaaaaa@37A7@ by its estimated standard deviation, and by assumption of normality a *P *value is obtained.

**Figure 7:**
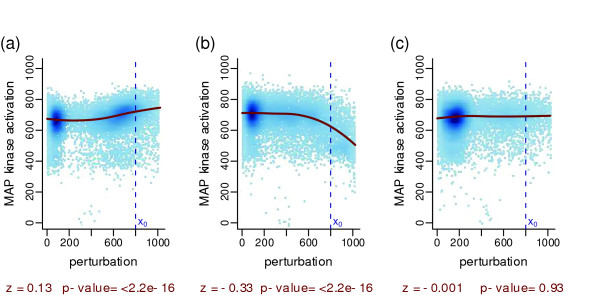
Example results for continuous responses from a MAPK screen. Effect size *z *and *P *value for **(a) **an activator (PLCD4), **(b) **a repressor (DUSP10), and **(c) **a non-affector (YFP) of the MAPK signaling. DUSP, dual specificity protein phosphatase; MAPK, mitogen-activated protein kinase; PLCD4, phospholipase C *δ*4; YFP, yellow fluorescent protein.

The plots in Figure 7 show the fitted local regression for three examples from a cell-based assay targeting the MAPK pathway. As a result of the overexpression of the phospholipase C *δ*4 (PLCD4) protein, our method detects a significant induction of extracellular signal-regulated kinase (ERK) activation (Figure 7a) - a finding that is consistent with previous reports [25]. As expected, overexpression of the dual specificity protein phosphatase (DUSP)10 protein strongly inactivates MAPK signaling (Figure 7b), whereas overexpression of the YFP protein has no effect (Figure 7c).

#### Summarizing replicate experiments

The *P *values obtained from the previous section test the statistical association between the fluorescence signals from the overexpressed YFP-tagged proteins and the reporter-specific antibodies for the cell population in one particular well. It is important to note that this only takes into account the cell-to-cell variability within that well and does not reflect higher levels of experimental and biologic variability. Hence, the results from a single well cannot simply be taken as a measure of biologic significance. To gain confidence in the biologic significance of a result, the next step is to consider measurements over several independently replicated wells.

The most obvious approach to summarizing data from replicate measurements for the same gene is to combine the effect size estimates and the *P *values from the individual replicates using tools from statistical meta-analysis [26]. However, because all of the data are available, the more direct and probably more efficient approach is to generalize the previous analysis methods and to deal with replicate wells. In particular, for stratified contingency tables in the case of binary responses, we use the stratified *Χ*^2^-statistic in the Cochran-Mantel-Haenszel test [27]. For stratified continuous responses we extend equation 3:

*y *= *y*_*i *_+ *m*(*x *- *x*_*i*_) + *ε*   (6)

Where *i *= 1, 2, ... counts over the replicates and x_i _and y_i _are replicate specific offsets. Again, in both cases we obtain estimates of effect size as well as significance.

#### Interpreting effect size and significance

Because of the large number of tests performed, it is necessary to adjust for multiple testing. Good software for this is available in the R packages qvalue and multtest, and we recommend the reports by Storey [28] and Pollard [29] and their coworkers for methodologic background.

Even after multiple testing adjustment, one will often encounter situations in which for many of the screened genes the null hypothesis of no effect will be rejected, although the effect sizes (equations 2 and 5) may be quite small for most of them. This can happen because of the large number of cells observed for each gene, and it is a well known phenomenon of statistical testing; when the number of data points becomes large, hypothesis tests will eventually reject any null hypothesis that differs from the truth, even in the most negligible manner [30]. Such cases are unlikely to be biologically interesting. Hence, for biologically relevant effectors we require both the effect size estimate to be above a certain threshold and the adjusted *P *value to be small.

Finally, as with any biologic assay, to corroborate conclusively the role of a protein in the cellular process of interest, independent validation experiments must be conducted according to best experimental practice.

### Visualization and quality assessment

Visualization methods exploit the most advanced pattern recognition system, the human visual system. However, it can only deal with a limited amount of dimensionality and complexity, and hence it benefits from assistance by computational methods for dimension reduction and feature extraction.

Here, our main focus is on the use of visualization for quality assessment, which for our kind of data must be done on three different levels: at the level of the individual well, with resolution down to data from individual cells; at the level of a microtiter plate, with resolution down to individual wells; or at the level of the gene of interest, which usually comprises several replicate experiments.

#### Visualization at the level of individual wells

**Figure 8:**
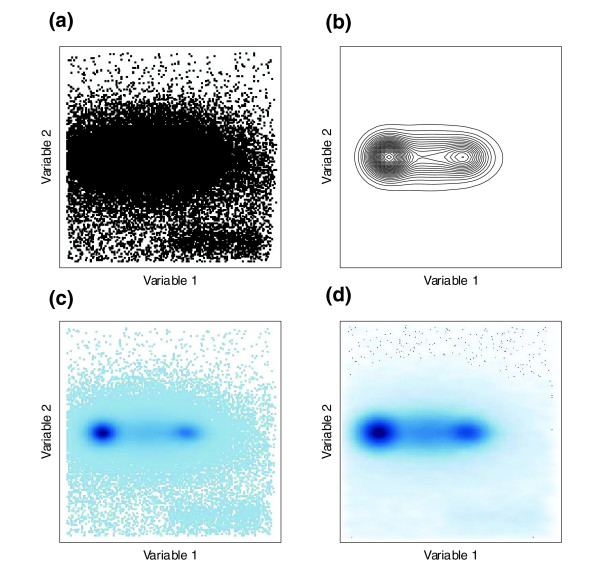
Options to create plots with high point densities. **(a) **Almost no features of the data distribution are visible in the simple scatter plot. **(b) **The contour plot reveals the bimodality of the data. **(c) **Coloring of points according to point density and **(d) **density map with additional points in sparse regions.

A simple but useful way to visualize bivariate data is by means of a scatterplot. However, it is difficult to get a good impression of the distribution of the data when the number of observations is large and the points become too dense (Figure 8a). This is a problem for cytometry data with often more than 20,000 data points. A way to circumvent this limitation (which has already been applied in some of the previous figures) is by plotting the densities of the data points at a given region [31] instead of individual points (Figure 8d) or, alternatively, by plotting each single point using a color coding that represents the density at its position (Figure 8c). We prefer false color coding to the commonly used contour plots (Figure 8b) because we find it more intuitive. By further augmenting false color density plots with outlying points, one can also visualize the data in sparse regions of the plot. We compute densities using a kernel density estimate.

#### Visualization at the level of microtiter plates

**Figure 9:**
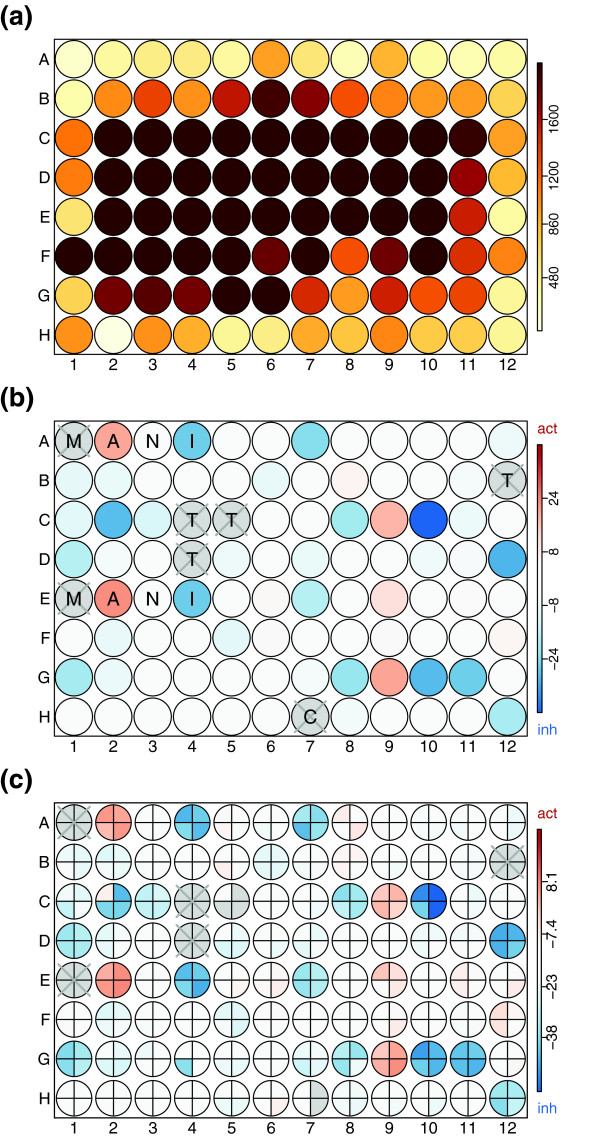
Plate plots show several aspects of the data in a format resembling a microtiter plate. This is useful for detecting spatial effects and to present concisely the data belonging to one experiment. **(a) **Quantitative values: number of cells in the well. The consistently lower number of cells at the edges of the plate indicate problems during cultivation. **(b) **Qualitative values: activators (red) and inhibitors (blue) of the process of interest. Wells that did not pass quality requirements are crossed out and wells containing cells treated with controls are indicated by capital letters. Cells in the first four rows of the plate were transfected with amino-terminally tagged expression constructs, and rows five to eight with carboxyl-terminally tagged constructs. **(c) **Comparison of results from four replicate plates. Each slice contains data from one replicate. Reproducibility between replicates is very high.

Most high-throughput applications in cell biology are carried out on microtiter plates which come in different formats, usually as a rectangular arrangement of 24, 96, 384, or 1536 wells. Each well may contain cells that have been treated in a different manner. An intuitive approach for visualization is to use the familiar spatial layout of the plate. Figure 9a shows an example of what we call a plate plot for a 96-well plate. It indicates the number of cells identified in each well. The consistently low number of cells on the edges of the plate suggests a handling problem, and subsequent analysis steps are possibly affected by this artifact. Other quantities of interest often include the average fluorescence of each well, for example to monitor expression efficiency or to detect artifactual shifts in the response.

Plate plots can also be used to present qualitative variables. Figure 9b shows the negative log transformed odds ratios from the statistical analysis of a 96-well plate from a cell proliferation assay. Negative values indicate inhibition of cell proliferation and are colored in blue, whereas positive values correspond to activation as indicated in red. The attention of the experimenter is immediately drawn to the few interesting wells and spatial regularities are easily spotted. In this example, we can compare the upper and lower halves of the plate; the top half contains cells transfected with carboxyl-terminally tagged constructs and the bottom half contains cell transfected with amino-terminally tagged constructs of the same genes. Additional information is added to the plot by using further formatting options, for instance crossing out of wells discarded from analysis or plotting additional symbols on wells with controls.

The amount of information included in a plate plot can be extended further by decorating it with tool tips and hyperlinks. When viewed in a browser, a tool tip is a short textual annotation, for example a gene name, that is displayed when the mouse pointer moves over a plot element. A hyperlink can be used to display more detailed information, even a graphic, in another browser window or frame. For example, underlying each value that is displayed in a plate plot such as Figure 9b is a complex statistical analysis, the details of which can be displayed on demand by hyperlinking them to the corresponding well icons in the plate plot. The reader is directed to the online complement [32] for an interactive example. Using plate plots in this way provides a powerful organizational structure for drill-down facilities because potentially interesting candidates are easily identified on a plate and the range of detailed information enables the experimenter to audit steps of the analysis procedure.

#### Gene centered visualization

**Figure 10:**
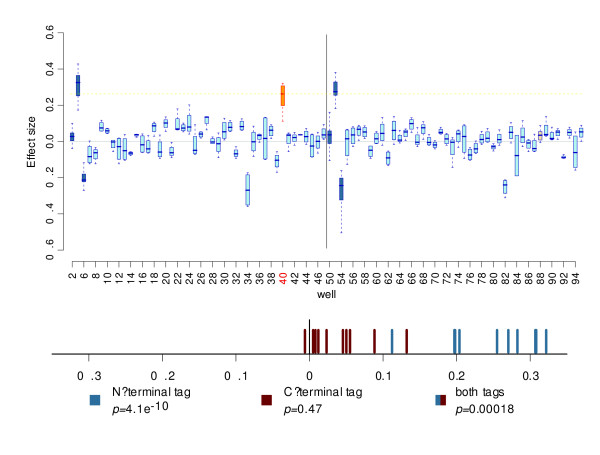
Interactive box plot of effect sizes from replicate experiments for a 96-well plate. Proteins showing consistently high or low effect sizes can easily be identified. By clicking on the individual boxes in the upper panel, a drill-down to the underlying data is provided in the lower panel, which shows the individual measurement values for both fluorescence tags as vertical bars along the x-axis. In this example, only the expression of the amino-terminally tagged protein results in significantly elevated effect sizes.

Because experiments are done in replicates, another level of visualization is needed to compare multiple measurements of the same gene over several plates. For a limited number of replicates the plate plot concept can be utilized. Besides colored circles, as in Figure 9 panels a and b, its implementation allows us to plot arbitrary graphs at each well position. In Figure 9c we use segmented charts to display the results from four replicate experiments (we call this a 'pizza plot'). For more extensive datasets, Figure 10 shows how hyperlinked box plots can be used to display multiple relevant aspects of the data. In this example they allow exploration of the effect of the orientation of the carboxyl-terminal or amino-terminal YFP fusion in the expression vectors.

### Application

**Figure 11:**
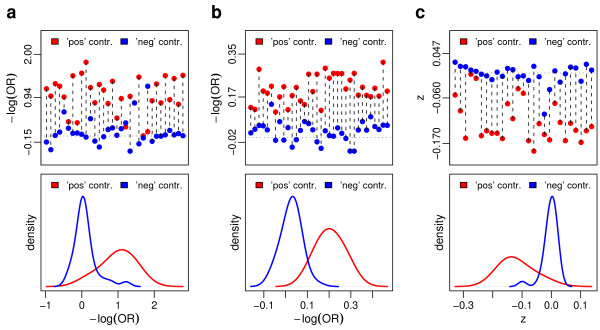
Separation of positive and negative controls. Top panels: effect sizes of positive and negative controls (y-axis) for individual plates (x-axis). Bottom panels: density plots of the joint effect sizes for controls across all plates. **(a) **Controls for the apoptosis assay are CIDE3 (positive) and CDK (negative). **(b) **Controls for the proliferation assay are cyclin A (positive) and YFP (negative). **(c) **Controls for the MAPK assay are DUSP10 (positive) and YFP (negative). The measured effect sizes for positive and negative controls separate well. CDK, cyclin-dependent kinase; DUSP, dual specificity protein phosphatase; MAPK, mitogen-activated protein kinase; YFP, yellow fluorescent protein.

We applied our method to the dataset introduced in the section Materials and methods (below) and verified the effects of positive and negative control genes of known function for each of the three assays with high specificity (Figure 11), thus validating the approach. The positive control for the apoptosis assay were vectors expressing CIDE3 (cell-death-inducing DFF45-like effector 3) and the Fas receptor, and the negative control were vectors expressing cyclin-dependent kinase and YFP. Positive and negative controls for the proliferation assay were vectors expressing cyclin A and YFP, respectively. In the MAPK assay, overexpression of DUSP10 was used as a positive control, and overexpression of YFP was used as a negative control. A total of 273 open reading frames (ORFs) encoding proteins of unknown function were selected based on cancer-associated alterations in their respective mRNA transcription. These ORFs were cloned in 546 amino-terminally as well as carobxyl-terminally fused expression constructs and were subsequently screened in the three assays. Eleven inhibitors and two activators of ERK phosphorylation were identified in the MAPK assay. The proliferation screen revealed four activators and five inhibitors. Eleven activators with significant effect on programmed cell death were identified in the apoptosis screen. For further details on these proteins, see Additional data file 1. The complete dataset is freely available from our web server [32].

## Conclusion

The increasing application of high-throughput technologies in cell biology has opened the way for systematic studies to be carried out on a large scale. This will allow us to gain an understanding of complex systems such as cellular pathways, because of the ability to measure the large number of parameters needed to model and reconstruct such systems (for instance, by combinatorial perturbations or time course experiments). However, the main prerequisite is a uniform, quantitative and comparable analysis of the raw data in order to integrate efficiently the information collected. Analyzing and managing the vast amount of data generated in these studies initially seems to be a daunting task.

Here, we show the complete work flow from raw flow cytometry data to a list of genes that are components of or interact with the cellular process of interest. Procedures (methodologic recommendations as well as software) for data pre-processing are presented that can be used to deal with typical sources of systematic variation. We stress the importance of monitoring crucial steps during analysis and show a range of visualization tools for quality control. Techniques are suggested to assess the data on different levels and to present results in a concise and meaningful way. By applying statistical methods, we are able to identify interesting phenotypes based on a set of objective criteria rather than relying on manual selections. Because data are available for each cell of a cell population, we are able to extract several kinds of information. Stratified statistical tests and models allow us to combine results from replicate experiments, further increasing precision.

To select genes of interest we consider two parameters, a threshold for the *P *value as well as one for the effect size. It is important to note that statistical significance and effect size are independent quantities, and that we must impose conditions on both of them if we are to obtain relevant results. In our screen the main focus lies on identifying candidates out of a pool of functionally unknown genes for further, in-depth analyses; thus, specificity is given preference over sensitivity, which is reflected in a rather conservative selection of threshold values.

Some of the methods described here are specific to flow cytometry measurements, but most of the visualization should also be applicable to data from other sources. Here we have only considered two simple models: binary and continuous responses. However, cell-based assays can be designed to assess almost any cellular process, and as the complexity of the observed phenotypes increase, so do the necessary statistical models. However, there will always be a need to summarize and simplify data to a form that is amenable to visual inspection and that allows for drill-down to more detailed aspects. In addition to specified analyses, we also wish to provide a framework that is easily adaptable and extendable to more complex assays and phenotypes.

All functionality is implemented using the statistical programming language R and is available as the software package prada through the open source Bioconductor project [19].

## Materials and methods

A total of 273 ORFs encoding proteins of unknown function were selected based on cancer-associated alterations in their respective mRNA transcription [33]. HEK 293T cells were transfected with expression constructs of the respective genes of interest fused to the YFP under the control of a cytomegalovirus promoter [34]. The amino-terminal or carboxyl-terminal fluorescence tags allowed us to monitor the level of expression along with the detection of induced effects. Cells were fixed 48 hours (MAPK and DNA replication assay) or 72 hours (apoptosis assay) after transfection and stained intracellularly with specific antibodies. Different antibodies were used for the different assays, each specifically measuring the phenotype of interest. In the case of cell proliferation, the antibody detected the incorporation of the thymidine analog BrdU into the replicated DNA. An antibody specific for the activated form of the caspase-3 apoptosis regulator was employed in the apoptosis assay; a phospho-specific antibody detecting phosphorylated ERK2 was used to measure activation of MAPK signaling. The same secondary antibody coupled to Allophycocyanin (APC) was used for immunostaining in all three assays. Flow cytometry data were acquired using an automated FACS instrument (BD FACS Calibur, Becton Dickinson Biosciences, 2350 Qume Drive, San Jose, Ca, USA).

## Additional data file

The following additional data are included with the online version of this article: The vignette of the accompanying R data package containing code samples and a more detailed description of the individual computational analysis steps, as well as tables of the candidates from our dataset identified in the three assays (Additional data file 1).

## Supplementary Material

Additional data file 1 The vignette of the accompanying R data package containing code samples and a more detailed description of the individual computational analysis steps, as well as tables of the candidates from our dataset identified in the three assays. Click here for file
