# Supplementary material for: Statistical methods and software for the analysis of highthroughput reverse genetic assays using flow cytometry readouts
Source: Genome Biol. 2006 Aug 17;7(8):R77. doi: 10.1186/gb-2006-7-8-r77 (PMC1779598; doi:10.1186/gb-2006-7-8-r77)
Supplement: Additional data file 1 — The vignette of the accompanying R data package containing code samples and a more detailed description of the individual computational analysis steps, as well as tables of the candidates from our dataset identified in the three assays. [file gb-2006-7-8-r77-S1.pdf]

Sample Analysis of cell-based screens

Florian Hahne

May 16, 2006

Contents

1 Introduction **1**

2 Apoptosis assay **2**
2.1 Data import and preprocessing 2
2.2 Single plate analysis 2
2.3 Combining replicates 2

3 Proliferation assay **3**

4 MAP-kinase assay **4**

5 List of Candidates **4**

6 Using cellHTS for downstream analyses **7**

7 Conclusion **8**

1 Introduction

In this vignette we will show a sample analysis for a data set generated in three cell-based assays screening for the effect of over-expression of unknown proteins on apoptosis, MAP-kinase activation and cell proliferation. We will present the complete workflow beginning from the raw data files to a list of candidates for one 96 well plate. We try to provide an audit trail that enables the reader to follow up individual steps of the analysis and to explore most of the visualization techniques used for quality assesment. All output will be interactive HTML and we will provide URLs where necessary to browse its contents. Many of the individual steps are done using predefined functions and the user is encouraged to take a look into their source code for a deeper understanding.

2 Apoptosis assay

We start with the analysis of the apoptosis assay. Some of the following steps have to be repeated for the other assays, i. e. data import and data preprocessing.

2.1 Data import and preprocessing

The complete data set is stored in a folder structure organized by assay type, screening plate and replicate. For each replicate well there is a set of FCS 2.0 files which contain the raw fluorescence measurements as produced by the BD FACSCalibur instrument. In addition, there are phenoData files, which essentially are text files and which contain the necessary meta data for each plate. Some diagnostic plots will already be produced at this stage, thus we need to provide the path to the output directory. Be aware that this directory will be created in the path in which this vignette is build. If you want to specify the output directory please change the value of the 'outBase' variable.

```
> outBase <- "."
> if (!file.exists(outBase)) dir.create(outBase, recursive = "TRUE")
> inBase <- system.file("extdata", "apoptosis", "plate1", package = "cbaDataDKFZ")
> outDir <- file.path(outBase, "apoptosis", "plate_1")
> dataSet <- preprocData(outBase = outBase, inBase = inBase)
```

2.2 Single plate analysis

Now that we have our data imported we can start the analysis. This step will produce some more plots and provide the HTML framework. All results are also saved in a text file ('results.txt') in the output directory. An entry point to the HTLM output can be found in the file 'analysis.html' in the apoptosis plate1 folder of the output directory

```
> setInfo <- read.table(file.path(inBase, "setInfo.txt"), header = TRUE,
+     sep = "\t", as.is = TRUE)
> results <- doAnApo(dataSet, setInfo, outBase)
```

2.3 Combining replicates

In a subsequent step we will combine the results from the four replicate plates to get a final list of candidates from the screen, with both information about effect size and significance. To have a more intuitive measure of effect size we take the negative log transformation of the odds ratio.

```
> final <- analyzeBinaryStrat(outDir)
> final$oddsRatio <- -log(final$oddsRatio)
> candidateList <- final[final$p.value < 0.01 & final$oddsRatio >
+     1.2, ]
> candidateList[order(candidateList$oddsRatio, decreasing = TRUE),
```

```
+     ]
> save(final, file = file.path(outDir, "apoFinal.Rd"))
```

3 Proliferation assay

We will now follow the same procedure as before to do the analysis of the proliferation assay, that is data import and preprocessing, creation of the audit trail and finaly output of a list of candidates.

Import:

```
> if (!file.exists(outBase)) dir.create(outBase, recursive = "TRUE")
> inBase <- system.file("extdata", "proliferation", "plate1", package = "cbaDataDKFZ")
> dataSet <- preprocData(outBase = outBase, inBase = inBase)
> outDir <- file.path(outBase, "proliferation", "plate_1")
> save(dataSet, file = file.path(outDir, "prolifDataSet.Rd"))
```

Single plate analysis:

```
> setInfo <- read.table(file.path(inBase, "setInfo.txt"), header = TRUE,
+     sep = "\t", as.is = TRUE)
> results <- doAnProlif(dataSet, setInfo, outBase)
```

Comparison of replicates:

```
> final <- analyzeBinaryStrat(outDir)
> final$oddsRatio <- -log(final$oddsRatio)
> candidateList <- final[final$p.value < 0.01 & abs(final$oddsRatio) >
+     0.2, ]
> candidateList[order(candidateList$oddsRatio, decreasing = TRUE),
+     ]
```

```
> if (file.exists(file.path(outDir, "prolifFinal.Rd"))) {
+     load(file.path(outDir, "prolifFinal.Rd"))
+     cat("data loaded from cache!\n")
+ } else {
+     final <- analyzeBinaryStrat(outDir)
+     final$oddsRatio <- -log(final$oddsRatio)
+     candidateList <- final[final$p.value < 0.01 & abs(final$oddsRatio) >
+         0.2, ]
+     candidateList[order(candidateList$oddsRatio, decreasing = TRUE),
+         ]
+     save(final, file = file.path(outDir, "prolifFinal.Rd"))
+ }
```

4 MAP-kinase assay

For this assay the first two steps are again the same.

Import:

```
> if (!file.exists(outBase)) dir.create(outBase, recursive = "TRUE")
> inBase <- system.file("extdata", "mapk", "plate1", package = "cbaDataDKFZ")
> dataSet <- preprocData(outBase = outBase, inBase = inBase)
> outDir <- file.path(outBase, "mapk", "plate_1")
> save(dataSet, file = file.path(outDir, "mapkDataSet.Rd"))
```

Single plate analysis:

```
> setInfo <- read.table(file.path(inBase, "setInfo.txt"), header = TRUE,
+     sep = "\t", as.is = TRUE)
> results <- doAnMap(dataSet, setInfo, outBase)
```

The additional step of producing a stratified data set is needed in order to compare between the plate replicates.

```
> dataSetStrat <- stratifyData(dataSet, outDir)
> final <- csApply(dataSetStrat, analyzeContStrat, simplify = TRUE)
> final <- cbind(pData(dataSetStrat)[, c("clone", "ORF", "localisation")],
+     t(final))
> final <- final[!is.na(final$delta), ]
> candidateList <- final[final$p.value < 0.01 & abs(final$zscore) >
+     3, ]
> candidateList[order(candidateList$delta, decreasing = TRUE),
+     -c(1, 5)]
```

5 List of Candidates

Final candidate lists including all replicates and plates have been included to this package as data files.

	Acc.Nr	Effect	pvalue	logOR	Gene.name
1	BC000255	act	0,000152	10,768	FUN14 domain containing 2
2	BC025382	act	3,3e-06	13,561	transketolase-like 1
3	AL136723	act	2,5e-05	84,94	chromosome 11 open reading frame 56
4	AL110297	act	0,00043	3,628	brain protein 44
5	AL136857	act	0,00195	8,579	solute carrier family 25 member 31
6	AL136781	act	0,0065	4,384	chromosome 3 open reading frame 20
7	AL136796	act	0,0054	11,308	kelch-like 25 (Drosophila)
8	BC015492	act	6,4e-07	8,778	interferon, alpha-inducible protein 27
9	BC009698	act	0,0085	7,335	apolipoprotein C-I
10	BC002503	act	2,8e-06	8,391	spermidine/spermine N1-acetyltransferase
11	AL136711	act	2,39e-52	12,283	transmembrane protein 49

Table 1: Apoptosis Assay

	Acc.Nr	Effect	pvalue	zscore	Gene.name
1	AL110297	inh	0,000259	22,729	brain protein 44
2	BC015492	inh	0,000323	18,65	interferon, alpha-inducible protein 27
3	AL136857	inh	0,000465	21,954	solute carrier family 25 member 31
4	BX648228	inh	5,03e-05	13,99	melanoma inhibitory activity 2
5	BC017713	act	0,29	15,135	CDC23 (cell division cycle 23, yeast, homolog)
6	BC040301	act	0,0001	17,005	PAS domain containing 1
7	AL834449	inh	0,00155	23,523	reticulon 4 receptor
8	AL136544	inh	6,01e-07	11,656	SMC6 structural maintenance of chromosomes 6-like 1
9	AL136891	inh	4,3e-08	15,86	NUAK family, SNF1-like kinase, 2
10	AL136832	inh	4,53e-07	14,966	Rho guanine nucleotide exchange factor (GEF) 3
11	AL136911	inh	0,000191	8,609	LIM domain and actin binding 1
12	BC032351	inh	0,00053	17,297	signal sequence receptor, delta
13	AL136761	inh	1,53e-05	15,778	radial spokehead-like 1

Table 2: MAP Kinase Assay

	Acc.Nr	Effect	pvalue	logOR	Gene.name
1	BM541828	act	0,0012	0,439	hypothetical protein LOC203547
2	BC025298	act	0,0132	0,331	interleukin 9 receptor
3	AL080177	act	0,0192	0,656	ubiquitin-like 3
4	AL136801	inh	1,4e-10	-3,374	transient receptor potential cation channel
5	AL110297	inh	0,00378	-1,694	brain protein 44
6	AL136891	inh	1,76e-06	-3,055	NUAK family, SNF1-like kinase, 2
7	BC000738	inh	2,83e-08	-3,253	emerin (Emery-Dreifuss muscular dystrophy)
8	AL136936	act	0,00607	1,476	heat shock 22kDa protein 8
9	BC002503	inh	2,05e-07	-4,329	spermidine/spermine N1-acetyltransferase

Table 3: Proliferaton Assay

6 Using cellHTS for downstream analyses

Although the package *cellHTS* is more geared towards analysis of unprocessed raw data we can still make use of its quality feedback and visualization features for our highly processed data. To exemplify the procedure we added some sample files for the apoptosis assay to this package which contain all the necessary information to be provided for *cellHTS* (it is straight forward but rather tedious to produce these files, so we did not reproduce this step here). For two replicates of two plates there are files containing the calculated odds ratios for each of the 96 wells. The file *Platelist.txt* maps these files to plates and replicates. By calling *readPlateData* we can import the data and generate a *cellHTS* object:

```
> experimentName = "ApoptosisScreen"
> dataPath = system.file("extdata", package = "cbaDataDKFZ")
> x = readPlateData("Platelist.txt", name = experimentName, path = dataPath,
+     plateType = "96")

Reading plate3_1.txt plate3_2.txt plate4_1.txt plate4_2.txt
Done.

> x

cellHTS object of name 'ApoptosisScreen'
2 plates with 96 wells, 2 replicates, 1 channel. State:
configured normalized     scored  annotated
     FALSE      FALSE      FALSE      FALSE
```

In a second step we tell *cellHTS* where to expect the controls and also give some details about the experiment. This information is provided by the files *Plateconf.txt*, *Screenlog.txt* and *Description.txt*.

```
> confFile = file.path(dataPath, "Plateconf.txt")
> logFile = file.path(dataPath, "Screenlog.txt")
> descripFile = file.path(dataPath, "Description.txt")
> x = configure(x, confFile, logFile, descripFile)
```

We ommit the normalization step since this has already been done during our analysis. However, we do need to tell *cellHTS* that this has been done in order to proceed to the following steps. We also want to calculate the negative log transformation of the odds ratio to ensure symmetry around zero.

```
> x$xnorm <- -log10(x$xraw)
> x$state["normalized"] <- TRUE
```

In the final step we include annotation information for both plates (provided by the file *GeneIDs* and generate the HTML report.

```
> geneIDFile = file.path(dataPath, "GeneIDs.txt")
> x = annotate(x, geneIDFile)
> writeReport(x, force = TRUE, plotPlateArgs = list(xrange = c(0.2,
+     1.5), xcol = c("white", "red")), imageScreenArgs = list(zrange = c(-2,
+     6.5), ar = 1))

[1] "/fsraid2/bioinf3/fctassays/cbapaper_web/build/ApoptosisScreen/index.html"
```

The final report can now be inspected in the subfolder *ApoptosisScreen* of the current working directory. For more information on each individual step please consult the vignette of the *cellHTS* package.

7 Conclusion

This short vignette is a guideline on how to perform our analysis on the data set included. Since most of the functionality is specific for our setting, data and experimental question it is not inculded in prada. However, in prada (and also in other Bioconductor/R packages) one can find all necessary building blocks and functions. The pieces of software in this package are only the glue that fits together the different bits and pieces. In our internal setting we have combined the analysis with database storage and also large parts of the output are generated as dynamic rather than static HTML.
